# Diurnal variation in physical activity and musculoskeletal injury risk across sex and habitual exercise timing among physically active students

**DOI:** 10.1038/s41598-026-56966-9

**Published:** 2026-06-06

**Authors:** Jarosław Domaradzki, Dawid Koźlenia

**Affiliations:** Department of Biological Principles of Physical Activity, Wroclaw University of Health and Sport Sciences, Wrocław, 51-612 Poland

**Keywords:** Circadian rhythm, Musculoskeletal health, Exercise, Health behavior, Injury, Sex, Risk assessment, Health care, Medical research, Risk factors

## Abstract

This study examined whether habitual exercise timing and sex are associated with lower-limb injury prevalence and frequency in physically active young adults. A total of 454 recreationally active university students (48% males; age = 22.1 ± 2.4 years) completed a validated survey assessing training exposure, experience, and injury history. Habitual physical activity timing was classified into three defined categories: morning (08:00–11:00), midday/afternoon (11:00–17:00), and evening/night (17:00–24:00). Group differences were examined using chi-square and log-linear analyses. Zero-inflated Poisson (ZIP) and multivariable logistic regression models were used to identify factors associated with injury counts and occurrence, adjusting for age, fat mass index (FMI), training load, and experience. Injury prevalence was higher in males than females (*p* = 0.048). Injury frequency differed across time-of-day categories (*p* = 0.004), with the highest burden among participants exercising evening/night. In ZIP models, males, exercising evening/night, and higher FMI were associated with higher injury counts, whereas older age was associated with fewer injuries. Logistic regression showed similar associations for injury occurrence (AUC = 0.74, 95% CI: 0.69–0.79). Lower-limb injury risk in young, physically active adults is consistently associated with habitual exercise timing, sex, and adiposity. These findings highlight the importance of considering exercise scheduling patterns and FMI in injury-prevention strategies.

## Background

Young adulthood represents a critical period for establishing lifelong physical activity (PA) habits, which profoundly shape long-term health outcomes. Maintaining regular physical activity during this stage is strongly associated with reduced cardiometabolic risk, improved mental health, and sustained participation in exercise across adulthood. However, musculoskeletal injuries represent one of the primary barriers to long-term physical activity adherence in young populations, as injury-related interruptions may lead to reduced participation or permanent withdrawal from exercise^1,2^. At the same time, this life stage is marked by heightened vulnerability to musculoskeletal injury owing to ongoing physiological maturation, hormonal fluctuations, and increasing training demands^3–5^. Epidemiological studies reveal that a substantial proportion of physically active young adults experience at least one musculoskeletal injury annually, with the lower extremities most commonly affected^6,7^. Beyond immediate biomechanical consequences, such injuries may negatively influence confidence, increase fear of re-injury, and disrupt the development of stable lifelong physical activity behaviors^8,9^.

Musculoskeletal injuries in active young adults arise from multiple interacting factors. Although previous research has primarily focused on training volume, intensity, and sport type, individuals exposed to comparable training loads frequently demonstrate markedly different injury outcomes, suggesting the involvement of additional modifiers such as physiological readiness, behavioral characteristics, and contextual factors including the timing of physical activity^10,11^. One such factor may be the timing of exercise relative to circadian regulation. Circadian rhythms influence physiological processes relevant to injury risk—including core temperature, muscle strength, reaction time, and fatigue tolerance^12,13^. These daily fluctuations are regulated by the central circadian pacemaker located in the suprachiasmatic nucleus, which synchronizes endocrine secretion, thermoregulation, sleep–wake cycles, and neural activation patterns across peripheral tissues^13^.

Core body temperature follows a pronounced circadian rhythm, typically reaching its nadir in the early morning and peaking in the late afternoon or early evening. Even relatively small temperature variations (~ 0.5–1.0 °C) may influence muscle-tendon stiffness, metabolic reaction rates, and nerve conduction velocity, thereby affecting force production and movement efficiency^12,13^. Similarly, cognitive and motor performance demonstrate time-of-day variability driven by circadian modulation of alertness and central information-processing capacity. Reaction time and attentional stability tend to improve across the day and decline under conditions of reduced arousal or accumulated wakefulness, potentially increasing movement error during complex motor tasks^8^. Circadian influences also extend to fatigue tolerance and perceived exertion, contributing to diurnal differences in exercise performance and motor control^7,13^.

Importantly, peak physiological performance does not necessarily coincide with reduced injury risk. While athletic performance commonly improves in the late afternoon^14^, evening exercise sessions may occur under accumulated physical and cognitive fatigue resulting from daily academic or occupational demands, whereas early-morning activity may take place under conditions of reduced physiological readiness or insufficient warm-up^12,15^. Experimental evidence further shows that postural stability varies across the day, with reduced stability during early-morning tasks^16^. Despite these mechanistic observations, direct epidemiological evidence linking habitual exercise timing with injury occurrence remains limited, particularly in non-elite young adult populations^17,18^.

In addition to physiological fluctuations, cognitive and psychological processes may further contribute to time-of-day differences in injury vulnerability. As daily demands accumulate, mental fatigue may impair attentional control, increase reaction-time variability, and reduce situational awareness, thereby compromising movement decision-making during dynamic tasks. Such mechanisms align with evidence linking cognitive fatigue and reduced executive functioning with musculoskeletal injury susceptibility.

Moreover, physiological readiness and injury susceptibility may not operate uniformly across sexes. Sex-related differences in hormonal regulation, fatigue responses, and recovery characteristics have been reported, alongside potential variation in circadian phase preference and daily performance responses^17,18^. These observations provide a theoretical basis to examine whether the relationship between habitual exercise timing and injury risk may differ between males and females.

To our knowledge, no previous study has examined the combined effects of habitual exercise timing, sex, body composition, training load, and training experience on injury risk specifically in young, physically active university students. Existing studies have typically investigated these determinants in isolation, and evidence integrating chronobiological, behavioral, and physiological factors within a single analytical framework remains scarce. By operationalizing time-of-day as a behavioral exposure and applying both logistic and zero-inflated Poisson (ZIP) modelling, this study provides a novel perspective on chronobiological and behavioral determinants of injury risk by examining both injury occurrence and injury counts.

Therefore, the primary aim of this study was to evaluate whether habitual exercise timing is associated with injury occurrence in physically active young adults while accounting for sex, body composition, training load, and training experience as potential modifying factors. Specifically, the objectives were to: (1) compare injury prevalence and counts between males and females across three aggregated time-of-day categories (morning (08:00–11:00), midday/afternoon (11:00–17:00), and evening/night (17:00–24:00)); (2) evaluate the sex × time-of-day association using log-linear analysis; (3) identify factors associated with injury occurrence using multivariable logistic regression models; and (4) assess whether including time-of-day improves predictive performance compared with models based solely on body composition, training load, and experience. The proposed hypotheses were grounded in the assumption that injury susceptibility may vary according to both chronobiological and behavioral factors^14^. Specifically, physiological readiness, fatigue tolerance, postural control, attentional capacity, and motor performance fluctuate across the day, which may alter the ability to tolerate physical load and maintain movement control during exercise^12,13^. In this context, evening/night exercise may coincide with accumulated physical and cognitive fatigue resulting from daily academic or occupational demands, whereas morning activity may occur under conditions of reduced physiological readiness^15^. At the same time, injury risk may be further modified by sex-related differences in physiological responses, adiposity-related mechanical loading, cumulative training exposure, and previous training experience^3,5^. Therefore, higher training load and lower training experience were expected to reflect greater exposure and potentially poorer load-management capacity. The study hypothesized that: (H1) injury prevalence and counts would be higher among participants habitually exercising in the evening/night; (H2) a significant interaction between sex and time of day would be observed; (H3) higher training load and lower training experience would be associated with increased injury risk; and (H4) inclusion of time-of-day would improve model prediction.

## Materials and methods

### Study design

This study used a cross-sectional design. This manuscript represents a secondary analysis of an existing dataset. The primary analysis, published previously, investigated the co-structure of physical activity and dietary patterns using multivariate statistical techniques. The present study focuses specifically on injury epidemiology, introduces a newly derived exposure variable (habitual time of day of physical activity), and applies a distinct analytical framework (log-linear modelling, zero-inflated Poisson regression, and multivariable logistic regression). Therefore, this analysis is methodologically independent and addresses a different set of research questions.

The study utilized a convenience sampling approach. Participants were recruited from students attending in-person classes at the university during the 2022–2023 academic year. All eligible students who met the inclusion criteria (physically active individuals participating in recreational sports) were invited to participate on a voluntary basis. This design is commonly applied in studies investigating ex-post (retrospective) injury occurrences. The predictor variables, including body composition and training characteristics, were assessed at the time of data collection, whereas the outcome variable—injury history—covered the 12 months preceding participation. This one-year retrospective window was selected to balance recall accuracy with the likelihood of capturing a representative number of injury events. The chosen time frame aligns with prior injury surveillance research in physically active populations and reflects both practical considerations (e.g., memory limitations) and methodological evidence indicating that a 12-month period provides sufficient data for reliable modeling without introducing substantial bias. The recruitment process and methodological background are detailed in previous publications^19^. The current analysis represents a secondary examination of the existing dataset^20^. The study investigated the relationship between time of day, physical activity load, and injury occurrence. Logistic regression models were used to estimate injury risk. Self-reported injury history served as the dependent variable, analyzed both as a binary outcome (injury present or absent) and as a count of injuries. Physical activity load and time of day were included as independent variables, along with their interaction. Sex and age were incorporated as covariates. This methodology facilitated a detailed evaluation of behavioral factors associated with injury risk in physically active university students.

Language editing: The English language of the manuscript was revised using an AI-based tool (ChatGPT, GPT-5, OpenAI, San Francisco, CA, USA) to improve clarity and grammar. The authors reviewed and edited the content as needed, and take full responsibility for the final version of the manuscript.

### Ethics

Ethical approval for the study was granted by the Senate Research Ethics Committee of Wroclaw University of Health and Sport Sciences (approval no. 13/2022). Prior to participation, all students received detailed information about the study procedures and provided their informed digital consent. All methods were performed in accordance with the relevant guidelines and regulations.

### Sample size

The sample size was determined using two complementary approaches. First, the commonly recommended rule of a minimum of 10 cases per predictor in regression models was considered to ensure stable odds ratio estimates^21–23^. Second, the required sample size was estimated using a formula for proportion estimates with a specified margin of error^24^. The general formula for estimating sample size at the 95% confidence level is:

$$\:\mathrm{n}=(\frac{1.96}{{\updelta\:}}{)}^{2}\times\:\mathrm{p}\left(1-\mathrm{p}\right)$$

where n is the required sample size, Z is the critical value for the 95% confidence level (Z = 1.96), p is the expected population proportion, and δ represents the accepted margin of error. Assuming *p* = 0.5 and δ = 0.05, the minimum required sample size was estimated as approximately 385 participants. To account for potential incomplete responses or dropout, this number was increased by 20%, resulting in a target sample of approximately 462 participants. For subgroups not meeting the conventional “10 cases per predictor” rule, this criterion was treated as a heuristic rather than a strict requirement. To address potential small-sample bias, a sensitivity analysis using Firth’s bias-reduced logistic regression was additionally performed^25^. The final analytical sample included 454 participants, which remained close to the calculated target sample size.

### Participants

The study population consisted of recreationally active university students aged 18–27 years who regularly participated in individual or team sports as part of their habitual physical activity. A total of 454 healthy students participated in the study, including 219 males (48%) and 235 females, enrolled during the 2022–2023 academic year. The proportion of males and females reflected the actual distribution within these study programs.

During the recruitment process, 715 students were initially assessed for eligibility. After screening, 261 individuals were excluded due to not meeting inclusion criteria (*n* = 73), declining participation (*n* = 56), incomplete data (*n* = 68), or other reasons (*n* = 64). The final analytical sample therefore consisted of 454 physically active students.

Participants represented both individual sports (*n* = 307) and team sports (*n* = 147). The composition of the sample was as follows: 130 males engaged in individual sports (including 22 in combat sports) and 89 males participating in team sports, as well as 177 females engaged in individual sports (including 22 in combat sports) and 58 females participating in team sports. Team sports included soccer, volleyball, basketball, and handball, which represent the most common recreational team sports practiced by students in the studied population. Preliminary inclusion criteria required participants to be physically active students attending in-person classes. Inclusion criteria were: (1) being a university student enrolled during the 2022–2023 academic year, (2) regular participation in physical activity or recreational sports, defined as a repeated pattern of participation in structured or recreational exercise, including individual or team-based activities, performed at least twice per week for a minimum of six months before data collection, and (3) attendance in in-person classes during the data collection period. Exclusion criteria comprised students involved in university-regulated competitive sports (e.g., members of official university teams competing in academic sport leagues) or students enrolled in specialized sport or elite performance programs involving structured competitive training and performance monitoring. Combat sports were practiced at a recreational level, as students involved in university-regulated competitive sports or elite performance programs were excluded from the study. In total, 454 students met the eligibility criteria and were included in the study.

### Data collection procedures

Data for the present study were collected using a combination of laboratory-based anthropometric measurements and a structured questionnaire assessing injury history and training characteristics. Anthropometric and body composition measurements were obtained during laboratory sessions, while injury history and behavioral variables were collected using a self-administered online questionnaire. The following subsections describe the measurement procedures and variables used in the analyses.

### Anthropometric and body composition measurements, asymmetry calculations

Anthropometric and body composition assessments were performed in the Biokinetics Research Laboratory, BLINDED. The facility operates under Quality Management System certifications PN-EN ISO 9001:2009 (Certificate No. PW-48606-10E) and PN-EN ISO 9001:2015 (Certificate No. PW-15105-22X).

Body height was measured twice to the nearest 0.1 cm using a GPM anthropometer (GPM Anthropological Instruments). Body weight and body fat mass were measured using a bioelectrical impedance analyzer (InBody230, InBody Co. Ltd., Cerritos, CA, USA). This device has demonstrated high reliability for body-composition assessment, including body fat percentage and fat mass, and has been previously compared with reference body-composition methods. Based on height, body mass, and body fat mass, the body mass index (BMI) and fat mass index (FMI) were calculated using the following formulas:

$$\:{\mathrm{BMI=}\frac{\mathrm{body\:mass}\text{}\left[\mathrm{kg}\right]}{\mathrm{body\:height}{\mathrm{\:[m}}^{\mathrm{2}}]}}^{}$$

$$\:{\mathrm{FMI=}\frac{\mathrm{body\:fat\:mass\:}\left[\mathrm{kg}\right]}{\mathrm{body\:height}{\mathrm{\:[m}}^{\mathrm{2}}]}}^{}$$

Because BMI and FMI capture overlapping aspects of adiposity, we a priori retained only one adiposity indicator in multivariable models to avoid collinearity. We compared two otherwise identical specifications including either BMI or FMI using AIC (Akaike Information Criterion)/BIC (Bayesian Information Criterion), discrimination (AUC – Area Under the Curve; with DeLong’s test), and calibration (Brier score, calibration intercept/slope) for logistic models, and AIC/BIC (with Vuong’s test for non-nested models) for ZIP models. The adiposity marker with superior overall model performance was retained for the primary analyses, and the alternative specification was examined as a sensitivity analysis, with the key findings reported in the Results section.

### Recording of musculoskeletal injuries

Injury data were collected using the validated Injury History Questionnaire (IHQ), whose reliability was previously confirmed with a Cronbach’s alpha coefficient of 0.836, indicating high internal consistency^26,27^. The IHQ captured the number and anatomical location of injuries sustained within the previous 12 months. For this study, analyses focused exclusively on lower-limb injuries. All injury data were self-reported, with participants independently completing the questionnaire via Google Forms. Data completeness was confirmed after merging survey responses with the physical measurement dataset. There were no missing IHQ data among the 454 participants included in the final analysis.

### Demographic and training load characteristics

In the present study, sex, age, body composition indices (BMI and FMI), training load, training experience, and habitual time of day of physical activity were treated as exposure variables potentially associated with injury risk. Independent variables included sex (males, females), age, body composition indices (BMI and FMI), training load, training experience and time of day of physical activity. Training load was quantified as the product of training frequency (days per week) and training duration (hours per session). Training experience, defined as the number of years of regular training, was also included as an independent variable in the models. The time of day for physical activity was initially classified into five hourly intervals: 08:00–11:00, 11:00–14:00, 14:00–17:00, 17:00–20:00, and 20:00–24:00. However, because some of these intervals included only a very small number of participants, which could compromise statistical power and model stability, the original categories were aggregated for subsequent analyses. Accordingly, physical activity timing was reclassified into three broader time-of-day categories based solely on clock time: morning (08:00–11:00), midday/afternoon (11:00–17:00), and evening/night (17:00–24:00). This aggregation was performed using a data-driven approach based on participant distribution and typical daily scheduling patterns observed in university populations, where physical activity is commonly organized before academic activities (morning), between daytime obligations (midday/afternoon), or after study and work commitments (evening/night). The adopted categorization therefore reflects habitual behavioral exposure rather than strict chronobiological phases. Importantly, merging adjacent time intervals ensured adequate subgroup sizes required for stable estimation in multivariable and zero-inflated regression models while preserving meaningful temporal distinctions in physical activity timing. The reported time of physical activity represents participants’ habitual training schedules and preferred time windows, rather than the exact timing of individual injury events.

The primary outcome variable was the occurrence of lower-limb injuries assessed using the Injury History Questionnaire (IHQ). The dependent variable - lower-limb injury occurrence - was analyzed both as the number of injuries (continuous count variable) and as the presence of at least one injury (binary variable: yes/no).

Although the Injury History Questionnaire collected information on injuries affecting all anatomical regions, the present study focused exclusively on lower-limb injuries. This restriction was applied to ensure methodological consistency across participants engaged in different types of sports. Lower-limb injuries represent the most common injury location in physically active populations and are strongly associated with fundamental locomotor activities such as running, jumping, and directional changes. In contrast, upper-limb and trunk injuries often arise from more heterogeneous mechanisms (e.g., contact events or accidental falls) that vary considerably across sports disciplines. Including these injury types in a single outcome could therefore introduce substantial variability and reduce interpretive clarity.

### Statistics

To enhance methodological transparency, the statistical workflow was organized to reflect a stepwise analytical structure, beginning with descriptive comparisons (sex and time-of-day patterns), followed by categorical association tests, and culminating in multivariable modelling. The rationale for each analytic choice—including the aggregation of time-of-day categories and the use of zero-inflated Poisson regression—was explicitly aligned with the distributional characteristics of the data and the theoretical framework of injury risk.

Before the main analyses, Shapiro-Wilk tests were conducted to assess the distribution of the data in continuous variables. Continuous variables were expressed as mean, standard deviation, and 95% confidence interval, while categorical variables were presented as numbers and frequencies. The numbers and percentages of students engaging in physical activity at different times of day, as well as the frequencies of injury occurrence, were both reported. The analyzed time-of-day categories included: morning (08:00–11:00), midday/afternoon (11:00–17:00), and evening/night (17:00–24:00), with analyses stratified by sex. The reported time of physical activity represents participants’ habitual exercise scheduling patterns and preferred training time windows, rather than the exact timing of individual injury events. Training load was operationalized as weekly exercise frequency multiplied by average session duration. While this metric reflects basic exposure volume, the dataset did not include intensity-based (e.g., heart rate, power output) or perceptual (e.g., RPE, session-RPE) indicators. Thus, the load measure should be interpreted as an approximate indicator of overall exposure rather than a comprehensive physiological load index.

**Fig. 1:**
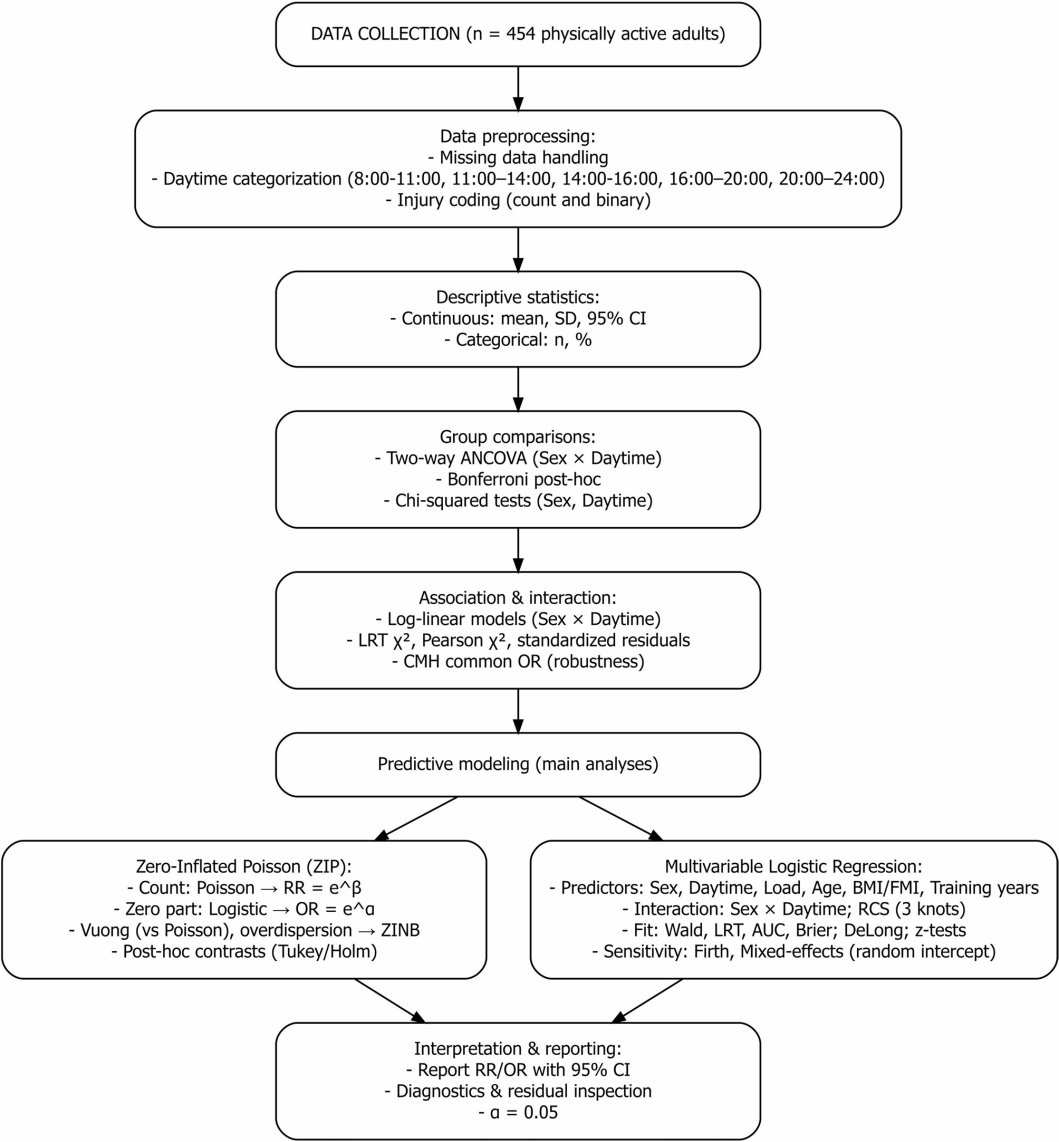
Analytical workflow of statistical procedures applied in the study. The figure illustrates the sequential structure of statistical analyses performed to examine injury risk and count in relation to daytime of physical activity, sex, and training load. The workflow includes data preprocessing, descriptive and comparative analyses, and two main predictive modeling approaches (Zero-Inflated Poisson and multivariable logistic regression).

Differences in continuous variables (age, body height, body weight, body fat percentage, body fat mass, BMI, FMI, training load, and training experience) between males and females were examined using an independent Student’s t-test. Differences between time-of-day categories were examined using two-way ANOVA, with sex and time of day (morning [08:00–11:00], midday/afternoon [11:00–17:00], and evening/night [17:00–24:00]) as factors. Detailed comparisons between categories were performed with Bonferroni-adjusted post-hoc tests. Differences in the frequency of injury occurrence between categorical variables (sex, daytime, body region) were tested using the chi-squared test with Yates correction. Effect sizes were expressed as φ coefficients, and odds ratios (OR) with 95% confidence intervals were calculated. To assess the association and interaction between sex and daytime in relation to injury counts at the group level, we fitted log-linear models to the aggregated contingency table. Evidence for interaction was tested using the likelihood ratio chi-square comparing the independence model to the interaction model. Model fit was evaluated with deviance, Pearson chi-square, and standardized residuals (with Bonferroni-adjusted residual diagnostics). For interpretability, we expressed effects as rate ratios derived from the Poisson parameterization (or equivalently as odds ratios when the table was formulated on injured vs. non-injured counts: Injury(yes/no) × Sex × Daytime). Where appropriate, we provided Cochran–Mantel–Haenszel common odds ratios stratified by daytime (or sex) as a robustness check. The main analyses consisted of two complementary modelling approaches. Zero-inflated Poisson (ZIP) regression was used for individual injury-count data, whereas multivariable logistic regression was used for the binary outcome of injury occurrence (yes/no). These models were applied because they address related but distinct aspects of injury epidemiology. The ZIP model estimates injury burden by modelling the number of injuries while accounting for the high proportion of participants reporting no injuries. In contrast, logistic regression estimates the likelihood of sustaining at least one injury, regardless of the number of injuries reported. Therefore, the combined use of these models allowed us to examine both injury frequency and injury occurrence within the same analytical framework. The model comprised a count component (Poisson with log link) and a zero-inflation component (logistic link). When modelling rates, a log-offset (e.g., person-time or an exposure proxy such as training load) was included. Model adequacy was evaluated via likelihood ratio tests (LRT) for nested specifications, Vuong’s test (ZIP vs. standard Poisson), and inspection of Pearson residuals. Model residuals were also visually inspected for systematic bias to verify distributional assumptions. In case of residual overdispersion, a zero-inflated negative binomial (ZINB) model replaced ZIP. We reported rate ratios (RR = e^β^) with 95% CI for the count component and odds ratios (OR = e^α^) with 95% CI for the zero-inflation component. Multiplicity-adjusted post-hoc contrasts (Tukey/Holm) of estimated marginal means were used to compare the eight sex-by-daytime groups on the count scale. Injury occurrence (1 = yes, 0 = no) was modeled using multivariable logistic regression. Fixed effects included sex, daytime of activity (morning (08:00–11:00), midday/afternoon (11:00–17:00), and evening/night (17:00–24:00)), and their interaction (sex × daytime), with age, BMI (or FMI), training years, and training load entered as covariates. For continuous covariates, potential non-linear associations on the logit scale were evaluated using restricted cubic splines. A 3-knot restricted cubic spline (Harrell’s method) was used to capture potential non-linear trends. Effect estimates were expressed as odds ratios (OR = e^β^) with 95% confidence intervals. Global effects and the interaction term were assessed using likelihood ratio and Wald tests. Discrimination and calibration were evaluated with the area under the receiver operating characteristic (ROC) curve (AUC), Brier score, and calibration intercept/slope; AUC differences between competing specifications were tested using the DeLong procedure. Model fit was considered acceptable for AUC ≥ 0.70 and Brier score ≤ 0.25. Multicollinearity was inspected via variance inflation factors. As sensitivity analyses, we fitted (i) Firth’s bias-reduced logistic regression to address sparse cells or (quasi) separation and (ii) mixed-effects logistic regression with a random intercept for cluster (e.g., sport or class) when applicable. Where class imbalance was substantial, models with inverse-probability class weights were additionally estimated. Marginal effects and pairwise contrasts among the eight sex-by-daytime groups were obtained from estimated marginal means with multiplicity adjustment (Tukey/Holm). Model performance and discrimination were further evaluated as follows: the goodness-of-fit and predictive accuracy of logistic models were assessed using Wald’s statistic, the Likelihood Ratio Test, and the area under the receiver operating characteristic curve (ROC). Differences in AUC values between models were compared using the DeLong test, while relative differences in predictive power between regression coefficients across models were examined using the z-statistic for β differences and for OR significance. Specifically, formulas were used:

$$\:{\mathrm{z=}\frac{{\text{}}_{1}-{\text{}}_{2}}{\sqrt{{\mathrm{S}\mathrm{E}}_{1}^{2}-{\mathrm{S}\mathrm{E}}_{2}^{2}}}}^{},\:\mathrm{a}\mathrm{n}\mathrm{d}\:{\mathrm{z=}\frac{\mathrm{l}\mathrm{o}\mathrm{g}\left({\mathrm{OR}}_{1}\right)-\mathrm{l}\mathrm{o}\mathrm{g}\left({\mathrm{OR}}_{2}\right)}{\sqrt{\mathrm{S}\mathrm{E}(\mathrm{l}\mathrm{o}\mathrm{g}{\mathrm{OR}}_{1}{)}^{2}-\mathrm{S}\mathrm{E}(\mathrm{l}\mathrm{o}\mathrm{g}{\mathrm{OR}}_{2}{)}^{2}}}}^{}$$

where:

z – is the z-statistic used to test the significance of the difference between regression coefficients (β) or odds ratios (OR) obtained from different models or groups; β – is the regression coefficient representing the effect of a given independent variable on the dependent variable (on the logit scale); SE – is the standard error of the regression coefficient, indicating the precision of the estimated β; log(OR) – is the natural logarithm of the odds ratio (equal to β in logistic regression); SE(log(OR)) – is the standard error of the log-transformed odds ratio, expressing the uncertainty of the estimate. Figure 1 presents analytical design. The significance level for all statistical tests and procedures was set at an α-value of 0.05. Calculations were conducted using Statistica 13.5 (StatSoft Poland 2023, Cracow, Poland) and RStudio 2025.05.0 + 496 (Posit Software, Boston, MA, USA).

## Results

### Basic descriptive characteristics of the males and females

Table 1 summarizes the descriptive characteristics of study participants, stratified by sex. Significant differences were observed between males and females in most anthropometric and training-related variables, including body height, body weight, body mass index (BMI), fat mass index (FMI), physical activity load, and training experience (*p* < 0.001). These differences confirm distinct profiles between sexes; therefore, sex was included as a key independent variable in all subsequent analyses.

**Table 1:** Baseline characteristics of participants by sex. P-values were obtained from independent-sample Student’s t-tests, with statistically significant differences highlighted in bold.

Sex			Males *N*=219			Females *N*=235			
Variable	**Mean**	**Lower** **95%CI**	**Upper** **95%CI**	**SD**	**Mean**	**Lower** **95%CI**	**Upper** **95%CI**	**SD**	**p-value**
Age [y]	21.8	21.5	22.0	1.9	21.2	21.0	21.4	1.5	**<0.001**
Body height [cm]	182.1	181.2	183.0	7.0	168.3	167.5	169.0	6.0	**<0.001**
Body weight [kg]	79.4	78.0	80.7	9.9	60.9	59.8	62.0	8.8	**<0.001**
Body fat [%]	16.1	15.5	16.7	4.5	23.3	22.6	24.0	5.5	**<0.001**
Body fat [kg]	12.9	12.3	13.5	4.5	14.6	13.9	15.3	5.4	**<0.001**
BMI [kg/m^2^]	23.9	23.6	24.2	2.5	21.5	21.1	21.8	2.7	**<0.001**
FMI [kg/m^2^]	3.9	3.7	4.1	1.4	5.1	4.9	5.3	1.8	**<0.001**
PA load [h/week]	6.3	5.7	6.8	3.9	5.6	5.2	6.1	3.8	0.093
PA experience [y]	3.6	3.4	3.8	1.3	3.1	2.9	3.3	1.5	**<0.001**

Injury prevalence differed across anatomical regions. Lower-limb injuries were the most frequent, occurring in 37.2% of participants, followed by upper-limb injuries (9.5%) and head/neck/trunk injuries (3.3%). When analyzed by injury type, joint sprains (24.5%) and muscle strains (17.8%) were the most common, followed by abrasions (13.2%) and fractures (4.2%).

Because the present study focuses specifically on lower-limb injuries, all subsequent analyses refer exclusively to lower-limb injury outcomes.

### Distribution of physical activity by sex and time of day

In the total sample of 454 participants, the vast majority reported engaging in physical activity in the evening/night (*n* = 360; 79.3%), while substantially fewer exercised in the midday/afternoon (*n* = 55; 12.1%) or in the morning (*n* = 39; 8.6%). This distribution indicates a clear predominance of evening physical activity. When stratified by sex, both males (*n* = 173; 38.1%) and females (*n* = 187; 41.2%) were most active in the evening (total: *n* = 360, 79.3%), whereas smaller proportions of males (*n* = 27; 6.0%) and females (*n* = 28; 6.2%) trained in the midday/afternoon (total: *n* = 55, 12.1%), and males (*n* = 19; 4.2%) and females (*n* = 20; 4.4%) trained in the morning (total: *n* = 39, 8.6%).

The distribution of males and females across the three time-of-day categories did not differ significantly (χ² = 0.02, df = 2, *p* = 0.988), with a negligible effect size (Cramer’s V = 0.007). Pairwise comparisons confirmed the absence of meaningful differences between sexes in specific time blocks: morning vs. evening/night (OR = 1.03, 95% CI: 0.53–1.99) and midday/afternoon vs. evening/night (OR = 1.04, 95% CI: 0.59–1.84). Thus, males and females exhibited comparable temporal patterns of physical activity.

### Basic descriptive characteristics of the males and females in PA time of a day categories

Table 2 summarizes the descriptive characteristics of study participants, stratified by sex. A two-way ANOVA was conducted to examine the effects of sex and time of day on physical activity, as well as their interaction, on key anthropometric and training-related variables. The analyses revealed no significant interactions between sex and time of day for any of the examined variables (all *p* > 0.05), indicating that sex-related differences were consistent across time-of-day categories.

**Table 2:** Descriptive morphological and training characteristics of males and females across physical activity time-of-day categories.

Statistics	Mean	Lower 95%CI	Upper 95%CI	SD	Mean	Lower 95%CI	Upper 95%CI	SD	Mean	Lower 95%CI	Upper 95%CI	SD
Variable	**M Morning**				**M Midday/Afternoon**				**M Evening/Night**			
Age [y]	20.3	18.2	23.8	1.1	21.8	19.1	25.5	1.8	22.0	18.6	27.8	1.9
Body height [cm]	182.3	170.1	193.1	7.3	181.9	168.2	191.8	7.1	182.1	163.1	197.9	7.0
Body weight [kg]	76.7	61.4	95.9	8.9	76.4	47.4	92.0	11.1	80.1	60.0	117.3	9.7
Body fat [%]	14.6	8.3	23.3	3.7	14.6	8.5	26.2	3.7	16.5	6.6	30.0	4.6
Body fat [kg]	11.4	5.1	20.8	3.9	11.3	5.6	18.4	3.6	13.3	4.3	30.3	4.6
BMI [kg/m^2^]	23.0	20.5	28.8	2.1	23.0	16.8	28.6	2.3	24.2	18.7	32.3	2.5
FMI [kg/m^2^]	3.4	1.7	6.7	1.1	3.4	1.8	5.3	0.9	4.0	1.4	8.9	1.4
PA load [h/week]	7.1	2.0	16.0	4.7	6.0	1.0	15.0	3.8	6.2	1.0	16.0	3.8
PA experience [y]	3.5	1.0	6.0	1.4	3.4	1.0	6.0	1.2	3.7	1.0	6.0	1.3
Variable	**F Morning**				**F Midday/Afternoon**				**F Evening/Night**			
Age [y]	20.9	19.5	24.4	1.4	20.9	18.0	24.3	1.2	21.3	18.6	26.1	1.6
Body height [cm]	167.5	153.0	179.7	6.5	169.3	160.0	185.4	5.7	168.2	153.4	184.5	6.1
Body weight [kg]	58.2	47.6	68.6	6.4	61.0	45.0	91.4	9.7	61.2	43.9	95.8	8.9
Body fat [%]	22.7	13.4	30.1	5.7	23.2	13.9	35.5	6.2	23.4	10.5	37.1	5.4
Body fat [kg]	13.5	6.5	20.1	4.5	14.6	6.2	32.5	6.3	14.7	4.9	35.8	5.4
BMI [kg/m^2^]	20.8	17.0	26.0	2.2	21.2	16.8	31.2	2.9	21.6	16.3	31.6	2.7
FMI [kg/m^2^]	4.8	2.5	7.5	1.6	5.0	2.4	11.1	2.0	5.2	1.7	11.5	1.8
PA load [h/week]	4.3	1.0	10.0	2.5	6.1	1.0	12.0	3.6	5.7	1.0	16.0	3.9
PA experience [y]	3.0	1.0	6.0	1.4	3.3	1.0	6.0	1.4	3.1	1.0	6.0	1.5

Significant main effects of sex were observed for body height (F = 214.0; *p* < 0.001; η²*p* = 0.192), body weight (F = 172.92; *p* < 0.001; η²*p* = 0.022), body fat percent (F = 117.79; *p* < 0.001; η²*p* = 0.072), body fat mass (F = 9.91; *p* = 0.002; η²*p* = 0.077), BMI (F = 35.16; *p* = 0.002; η²*p* = 0.072), FMI (F = 37.13; *p* < 0.001; η²*p* = 0.009), and PA experience (F = 4.11; *p* = 0.043; η²*p* = 0.009). These findings confirm that males and females differ significantly in these characteristics, regardless of the time of day when physical activity occurred. Overall, there was no evidence that time of day modified sex-related differences in these variables.

### Association Between Sex, Time of Day of Physical Activity, and Injury Occurrence

To aid interpretation, the key findings from the categorical analyses are summarized here. Injury prevalence differed by time of day, with the highest rates observed among evening exercisers and the lowest among morning and midday groups. Males exhibited a higher overall prevalence of injuries than females. However, contrary to our hypothesis, the sex × time-of-day interaction was not significant: the temporal pattern of injury risk was similar in both sexes. Thus, sex and time of day contributed independently to injury occurrence. Contingency Table 3 presents the distribution of individuals with and without injuries across sex and time-of-day categories. The proportions of participants in each group are indicated, allowing for a clear comparison of injury occurrence between males and females at different times of day. The relationships between these variables were further analyzed using loglinear analysis.

**Table 3:** Contingency table showing the number and percentage of participants with and without injuries by sex and time of day of physical activity.

Sex	Time of Day	Injury	Count	% of total
	Morning	Injury	14	3.1%
		No injury	5	1.1%
Male	Midday/Afternoon	Injury	13	2.9%
		No injury	14	3.1%
	Evening/Night	Injury	94	20.7%
		No injury	79	17.4%
	Morning	Injury	12	2.6%
		No injury	8	1.8%
Female	Midday/Afternoon	Injury	16	3.5%
		No injury	12	2.6%
	Evening/Night	Injury	79	17.4%
		No injury	108	23.8%

Among males, the largest absolute contribution to the total number of injury cases was observed in those who exercised in the evening/night (20.7% of the total sample), whereas fewer injury cases were observed among those exercising in the midday/afternoon (2.9% of the total sample). Among females, the largest absolute contribution to the total number of injury cases was also observed in the evening/night group (17.4% of the total sample), whereas fewer injury cases were observed among those exercising in the morning (2.6% of the total sample). At the onset of the log-linear analysis, the model was specified with female and morning hours as reference categories, and injury occurrence (yes) as the target outcome. An omnibus test was performed to determine the most parsimonious model explaining the joint distribution of Sex, Time of Day, and Injury. A loglinear analysis revealed significant main effects for both sex (LRχ² = 6.2, df = 1, *p* = 0.013) and time of day (LRχ² = 25.2, df = 2, *p* < 0.001) on injury risk. The best-fitting model included the Sex × Injury and Time × Injury interactions (χ² = 2.25, *p* = 0.324), indicating that the overall structure adequately represented the observed data. Partial chi-squared tests revealed a significant Sex × Injury association (χ² = 4.29, *p* = 0.038), suggesting higher injury prevalence among males, and a near-significant trend for Time × Injury (χ² = 5.12, *p* = 0.077).

The Sex × Time association was not significant (χ² = 0.03, *p* = 0.986), confirming that the temporal distribution of physical activity did not differ substantially between males and females. To verify robustness, stratified odds ratios were computed using the Cochran–Mantel–Haenszel (CMH) method. The CMH test showed a borderline overall association between sex and injury occurrence after controlling for time of day (common OR = 1.42; 95% CI = 0.92–2.20; χ² = 3.06; *p* = 0.081), while the Breslow–Day test indicated that the strength of this association was consistent across time-of-day strata (χ² = 1.02; df = 2; *p* = 0.60).

Together, these results confirm that both sex and time of day independently contribute to injury occurrence, with no evidence of a modifying interaction between them.

Because the categorical analyses do not capture continuous predictors (training load, training experience, body composition), we next examined these factors using zero-inflated Poisson and multivariable logistic regression models to address injury counts and injury occurrence, respectively.

### Predictors of injury risk and frequency: zero-inflated poisson and logistic regression analyses

Before including adiposity measures in the multivariable models, we first checked how both body mass index (BMI) and fat mass index (FMI) predicted injury risk, while accounting for sex and the time of day when physical activity occurred. In these adjusted logistic regressions, both indices were associated with a higher risk of injury; however, the model using FMI performed slightly better. Specifically, FMI resulted in a lower AIC (452.1 vs. 456.3) and a higher AUC (0.713 vs. 0.684) compared to BMI, while their calibration was similar (Brier score = 0.202 vs. 0.209). The regression coefficient for FMI was statistically significant (β = 0.184, SE = 0.072, *p* = 0.012; OR = 1.20, 95% CI: 1.04–1.39), indicating that higher FMI was associated with a greater injury risk. In contrast, the effect of BMI was not significant (β = 0.056, SE = 0.047, *p* = 0.23; OR = 1.06, 95% CI: 0.96–1.17). Because FMI provided better model performance and had a stronger association with injury risk, we used FMI as the primary measure of adiposity in all subsequent ZIP and multivariable logistic regression models. BMI was excluded from the final analyses to avoid collinearity; however, it was re-tested in additional sensitivity models, which yielded consistent results.

### Zero-inflated poisson regression for injury counts

The distribution of injury counts displayed a large proportion of zeros, far exceeding what would be expected under a standard Poisson process. This pattern is theoretically consistent with two distinct subpopulations: individuals who were effectively not at risk during the observation window (structural zeros) and individuals who were at risk but did not experience an injury (sampling zeros). Because this mixture violates the assumptions of ordinary Poisson regression, a zero-inflated Poisson model was selected to simultaneously estimate (i) the probability of belonging to the always-zero group and (ii) the expected number of injuries among those at risk. The number of injuries was analyzed using a zero-inflated Poisson (ZIP) regression model to account for the excess of zero counts. The model included sex, daytime of physical activity, and their interaction, with age, FMI, training load, and training experience as covariates. The detailed results of the zero-inflated Poisson regression model, including rate ratios (RR) for the count component and odds ratios (OR) for the zero-inflation component, are presented in Table 4. The ZIP model provided a better fit than the standard Poisson model, as shown by lower AIC (1523.7 vs. 1608.0), lower BIC (1589.8 vs. 1667.6), and a higher log-likelihood (–748.9 vs. − 793.0). In the count component of the model, males had a higher expected number of injuries than females (RR = 1.32, 95% CI 1.09–1.60, *p* = 0.004). Higher FMI was associated with a higher number of injuries (for every 1 kg/m² increase: RR = 1.06, 95% CI 1.01–1.11, *p* = 0.021), and older age was associated with fewer injuries (for each extra year: RR = 0.91, 95% CI 0.86–0.97, *p* = 0.001). Training load and experience did not show significant effects (both *p* > 0.40). In the zero-inflation part of the model, being active in the morning was associated with higher odds of being in the group that reported no injuries at all (the “always-zero” group; OR = 2.02, 95% CI 1.01–4.06, *p* = 0.047), while higher weekly training load slightly reduced those odds (for each extra hour per week: OR = 0.96, 95% CI 0.93–0.99, *p* = 0.006). This means that students who exercised in the morning were approximately twice as likely to belong to the subgroup that did not report any injuries during the study period.

**Table 4:** Results of zero-inflated Poisson regression model predicting the number of injuries. The count component represents the expected number of injuries (rate ratios, RR = eβ), while the zero-inflation component models the probability of belonging to the “always-zero” subgroup (odds ratios, OR = eα). Reference categories: Female (sex) and Evening/Night (time of day). Covariates included age, fat mass index (FMI), training load, and training experience.

Predictor	β (SE)	RR / OR	95% CI	*p*-value	Component
Intercept	2.64 (0.60)	14.01	4.27–45.93	<0.001	Count
Sex (Male)	0.28 (0.10)	1.32	1.09–1.60	0.004	Count
Time of day: Morning (vs. E/N)	–0.03 (0.13)	0.97	0.77–1.22	0.806	Count
Time of day: Midday/Afternoon (vs. E/N)	–0.26 (0.13)	0.77	0.59–1.01	0.058	Count
FMI (per 1 kg/m²)	0.06 (0.03)	1.06	1.01–1.11	0.021	Count
Age (per 1 year)	–0.10 (0.03)	0.91	0.86–0.97	0.001	Count
Training load (h/week)	0.00 (0.00)	1	0.99–1.01	0.807	Count
Training experience (years)	0.02 (0.03)	1.02	0.96–1.09	0.434	Count
Sex (Male)	–0.25 (0.18)	0.78	0.54–1.13	0.162	Zero-inflation
Time of day: Morning (vs. E/N)	0.70 (0.35)	2.02	1.01–4.06	0.047	Zero-inflation
Time of day: Midday/Afternoon (vs. E/N)	0.26 (0.31)	1.29	0.70–2.37	0.411	Zero-inflation
FMI (per 1 kg/m²)	–0.03 (0.04)	0.97	0.90–1.04	0.417	Zero-inflation
Age (per 1 year)	–0.02 (0.03)	0.98	0.93–1.04	0.601	Zero-inflation
Training load (h/week)	–0.04 (0.02)	0.96	0.93–0.99	0.006	Zero-inflation
Training experience (years)	0.02 (0.04)	1.02	0.95–1.09	0.654	Zero-inflation

Footnote: RR – rate ratio; OR – odds ratio; SE – standard error; CI – confidence interval. Rate ratios (RR = e^β^) represent multiplicative effects on the expected number of injuries (count component). Odds ratios (OR = e^α^) represent multiplicative effects on the probability of belonging to the zero-inflated (“no injury”) subgroup. Reference categories: Female (sex), Evening/Night (E/N). Covariates included age, fat mass index (FMI), training load, and training experience.

### Effect of predictors on the occurrence of the injury – multivariable logistic regression results

Injury occurrence (1 = yes, 0 = no) was modeled using multivariable logistic regression with the same set of predictors and covariates. The models were evaluated for overall fit, discrimination (AUC), and calibration, with additional robustness checks using Firth’s bias-reduced logistic regression where applicable. The detailed model estimates are presented in Table 5.

To identify predictors of injury occurrence, we fitted a multivariable logistic regression using the same set of variables as in the ZIP analysis. The overall model showed acceptable fit (χ² = 41.2, *p* < 0.001; Nagelkerke R² = 0.218), and demonstrated acceptable discrimination between injured and non-injured participants (AUC = 0.742, 95% CI: 0.696–0.783; Brier score = 0.182). Among the different factors, both sex and time of day were significant predictors. Males were more likely to report an injury than females (OR = 1.87, 95% CI: 1.28–2.73, *p* = 0.001). Compared to participants who exercised in the evening/night, those who trained during midday/afternoon were less likely to report an injury (OR = 0.64, 95% CI: 0.41–0.98, *p* = 0.041), while morning hours did not differ significantly from evening/night activity (OR = 0.92, 95% CI: 0.55–1.54, *p* = 0.745). Higher fat mass index (FMI) was associated with a higher risk of injury (per 1 kg/m²: OR = 1.14, 95% CI: 1.04–1.26, *p* = 0.006), while older participants were less likely to report an injury (per year: OR = 0.91, 95% CI: 0.85–0.97, *p* = 0.004). Training load and experience did not have a significant effect (both *p* > 0.20). These findings were consistent in additional analyses using Firth’s bias-corrected logistic regression, which supports that the results are robust.

**Table 5:** Results of multivariable logistic regression predicting injury occurrence (yes/no).

Predictor	β (SE)	OR	95% CI	*p*-value
Intercept	–1.28 (0.42)	0.28	0.12–0.63	0.002
Sex (Male)	0.63 (0.19)	1.87	1.28–2.73	0.001
Time of day: Morning (vs. E/N)	–0.08 (0.27)	0.92	0.55–1.54	0.745
Time of day: Midday/Afternoon (vs. E/N)	–0.45 (0.22)	0.64	0.41–0.98	0.041
FMI (per 1 kg/m²)	0.13 (0.05)	1.14	1.04–1.26	0.006
Age (per 1 year)	–0.10 (0.03)	0.91	0.85–0.97	0.004
Training load (h/week)	0.01 (0.01)	1.01	0.99–1.03	0.262
Training experience (years)	0.02 (0.04)	1.02	0.94–1.10	0.512

Footnote: OR – odds ratio; SE – standard error; CI – confidence interval. Reference categories: Female (sex) and Evening/Night (time of day); Covariates included age, fat mass index (FMI), training load, and training experience. Model performance: χ² = 41.2, *p* < 0.001; Nagelkerke R² = 0.218; AUC = 0.742 (95% CI: 0.696–0.783); Brier score = 0.182.

## Discussion

The novelty of the present study lies in examining how habitual exercise timing across three aggregated time-of-day categories is associated with sex, adiposity, training load, and experience in relation to injury risk within a young, physically active university population. Importantly, the Results demonstrated that male sex, higher FMI, and habitual evening/night exercise were consistently associated with greater injury burden across all applied analytical models, while morning or midday activity and older age showed protective associations. Half of the participants experienced at least one musculoskeletal injury, with lower-limb injuries being the most prevalent, directly supporting the epidemiological relevance of the investigated problem.

With regard to the hypotheses stated in the Introduction, the findings supported H1, as participants habitually exercising in the evening/night showed greater injury occurrence and higher injury counts. H2 was not supported, because the sex × time-of-day interaction was not significant, indicating that temporal patterns of injury risk were generally comparable between males and females. H3 was only partially supported, as neither higher training load nor lower training experience remained a significant predictor in the final multivariable models, although adiposity and other covariates contributed to injury risk. H4 was partly supported, because the inclusion of time-of-day improved the interpretation of injury risk and contributed to model performance. Overall, these findings suggest that habitual exercise timing contributes independently to injury risk rather than amplifying sex-related differences, which is consistent with the log-linear and CMH analyses. Similar independent contributions of exposure-related and sex-related factors have been previously reported in both sport and occupational settings^34,35,38,39^.

Consistent with model outcomes, habitual evening/night exercise timing was associated with greater injury occurrence and higher injury counts, whereas midday/afternoon activity was associated with lower odds of injury occurrence. However, habitual exercise timing in the present study reflected participants’ usual behavioral pattern of physical activity, namely their regular training schedule, and not the exact time at which injuries occurred. Accordingly, the observed associations should be interpreted as relationships between regular exercise scheduling patterns and retrospectively reported injury outcomes, rather than as evidence that injuries occurred during morning, midday/afternoon, or evening/night sessions. Given the observational and retrospective design, these findings should not be interpreted causally. From a physiological perspective, accumulated fatigue throughout the day and shortened recovery before sleep may impair motor control and decision-making in evening sessions^4,5,36^. Although physical performance often peaks later in the day due to circadian influences^14^, elevated performance capacity does not necessarily translate into lower injury risk when cognitive fatigue and external demands are simultaneously increased^6,7,37^.

Body composition analyses further showed that FMI provided stronger discrimination of injury risk than BMI, as reflected by lower AIC and higher AUC values. In practical terms, a higher FMI indicates a greater amount of fat mass relative to body height and may therefore reflect a less favorable body-composition profile for repeated loading of the lower limbs. In the present study, FMI was used as a continuous indicator rather than a categorical diagnostic marker, because recognised FMI cut-off values for classifying lower-limb injury risk in physically active young adults are not available^30^. Therefore, FMI should not be interpreted as a stand-alone screening threshold, but rather as an adiposity-specific measure that may help identify individuals who could require closer monitoring of training exposure, load progression, and recovery. Increased adiposity may elevate mechanical stress on joints, alter movement mechanics, and promote inflammatory responses following exercise-induced tissue stress^28–32^. Although previous studies have demonstrated associations between BMI and injury risk^33^, comparisons directly incorporating FMI remain limited, supporting the relevance of adiposity-specific indicators in injury epidemiology.

Psychological mechanisms may additionally explain the elevated injury risk observed during evening activity. Sustained wakefulness and accumulated academic or occupational demands may reduce attentional control, increase reaction-time variability, and impair executive functioning, thereby increasing movement-error probability during dynamic tasks^14,37^. These mechanisms align with the observed temporal injury patterns reported in the Results section, suggesting that injury vulnerability reflects combined physiological and cognitive loading rather than performance capacity alone^38,39^.

Both log-linear and CMH analyses confirmed higher injury prevalence among males alongside a borderline effect of time of day, without interaction between factors. This indicates independent rather than synergistic contributions of sex and exercise timing to injury occurrence, supporting prevention strategies that address exposure management and scheduling separately^8,40^.

The zero-inflated Poisson model demonstrated superior fit compared with the standard Poisson model and showed that male sex, higher FMI, and evening training increased injury counts, whereas older age reduced injury occurrence^41^. As discussed, this association may reflect greater training experience and improved load regulation with age, although a selection effect cannot be excluded whereby injury-prone individuals reduce long-term participation^40^.

The zero-inflation component further indicated that morning activity and lower weekly training load increased the probability of remaining injury-free, consistent with previous observations of protective moderate training exposure^42,43^. The agreement between ZIP and logistic regression findings strengthens confidence in the robustness of the identified predictors^3,8^.

From a practical perspective, the results suggest that habitual training timing may represent a relevant behavioral factor associated with injury occurrence. However, because causal inference is not possible from the present design, these findings should be interpreted cautiously^44,45^. Whenever feasible, individuals who habitually train in the evening/night may benefit from greater attention to warm-up procedures, fatigue monitoring, and progressive load management^8^. Incorporating FMI as a continuous adiposity-related indicator into routine assessments may further assist in identifying individuals with relatively higher fat mass in relation to body height, who may require more individualized progression strategies and closer monitoring of training exposure^3,30,46,47^. However, because no injury-specific FMI cut-off values have been established for this population, FMI should be interpreted comparatively and in combination with other clinical, anthropometric, and training-related information rather than as a stand-alone threshold-based screening tool.

Several limitations should be acknowledged. The retrospective and self-reported nature of injury and exposure data introduces potential recall bias, particularly regarding activity timing and injury history. Moreover, habitual exercise timing was assessed as a behavioral exposure, whereas the exact timing of injury occurrence was not recorded. Consequently, the present design does not allow conclusions regarding whether injuries occurred during morning, midday/afternoon, or evening/night exercise sessions. Thus, the time-of-day variable should be understood as a marker of regular exercise habits rather than as a direct temporal marker of injury events. Aggregation of time-of-day categories, although necessary to avoid sparse-cell artefacts, reduced temporal resolution. In addition, the distribution of participants across time-of-day categories was imbalanced, with most participants reporting evening/night activity and substantially fewer participants classified in the morning or midday/afternoon groups. This imbalance may have reduced statistical power for comparisons involving less represented categories and may have limited the precision of estimates for morning and midday/afternoon activity. Training load calculated as frequency × duration did not capture exercise intensity or internal load variability. Additionally, the physically active university sample may limit generalizability beyond similarly active populations. Another limitation is that weight-control strategies commonly used in competitive combat sports (e.g., rapid weight loss before competitions) were not assessed. However, participants were recreationally active students and individuals involved in competitive sport programs were excluded, which likely minimized the occurrence of such practices. Moreover, several potential sources of bias should be acknowledged. First, the use of a convenience sample of physically active university students may introduce selection bias and limit the generalizability of the findings to other populations. Second, injury data were self-reported, which may introduce recall bias; however, the recall period was restricted to 12 months, a time frame commonly used in epidemiological studies to improve reporting accuracy. Third, although anthropometric measurements were obtained using standardized laboratory procedures and validated instruments, measurement bias cannot be completely excluded. Despite these limitations, the consistent findings across analytical approaches indicate that habitual exercise timing, adiposity, and sex represent independent contributors to injury risk in young adults. Future studies should incorporate objective monitoring of training intensity, sleep, and physiological readiness to determine whether modifying exercise timing can causally reduce injury occurrence. Together, these findings highlight that injury prevention strategies in young adults should consider not only training load but also daily scheduling patterns, accumulated fatigue, and body-composition characteristics when designing training programs.

## Conclusion

This study showed that retrospectively reported lower-limb injury occurrence in young, physically active adults was associated with sex, body composition, and habitual exercise timing. However, habitual exercise timing reflected usual training schedules rather than the exact timing of injury events, and causal conclusions cannot be established from the present observational and retrospective design. Males, individuals who habitually exercised in the evening/night, and those with a higher fat mass index (FMI) were more likely to experience injuries, while exercising in the morning or midday/afternoon and being older were associated with a lower injury risk. Although both sex and exercise timing were significantly associated with injury occurrence, their effects operated independently. The hypothesized sex × time-of-day interaction (H2) was not confirmed, indicating that patterns of injury risk across the three aggregated time-of-day categories were similar in males and females. These findings highlight the value of considering habitual exercise scheduling, using FMI as an indicator of individual risk, and carefully managing training loads to support safer participation and injury-prevention strategies.

## Data Availability

The datasets used and/or analysed during the current study are available from the corresponding author on reasonable request.
